# Response to: RSBMT-2018-0270.R2 - Pain and quality of life in human T cell lymphotropic virus type 1 associated myelopathy or tropical spastic paraparesis after home-based exercise protocol: Randomized clinical trial

**DOI:** 10.1590/0037-8682-0723-2021

**Published:** 2022-06-06

**Authors:** Maíra Carvalho Macêdo, Renata de Sousa Mota, Naiane Araújo Patrício, Abrahão Fontes Baptista, Antônio de Souza Andrade, Katia Nunes Sá

**Affiliations:** 1 Escola Bahiana de Medicina e Saúde Pública, Programa de Pós-graduação em Medicina e Saúde Humana, Salvador, BA, Brasil.; 2 Universidade Federal do Recôncavo da Bahia, Curso de Engenharia em Tecnologia Assistiva e Acessibilidade, Cruz das Almas, BA, Brasil.; 3 Universidade Federal da Bahia, Programa de Pós-graduação em Medicina e Saúde, Salvador, BA, Brasil.; 4 Universidade Federal do ABC, Programa de Pós-graduação em Neurociência e Cognição, São Bernardo do Campo, SP, Brasil.

We would like to thank the reviewers for their comments regarding the possibility of the noninclusion of patients with psychiatric and neurological comorbidities in our study. This would have been very interesting, but it would be almost impossible to conduct such an investigation. Regarding the noninclusion of patients with neurological conditions, this would not apply to a sample with associated myelopathy (HAM/TSP), as this is a neurological condition *per se*. The noninclusion of patients with comorbid psychiatric symptoms would have helped answer the question of the efficacy of an exercise protocol in the control of pain, but around 70% of our symptomatic patients with HAM/TSP present depression traits as comorbidity^1^. Hence, their exclusion would substantially decrease the sample size and prevent the study from being developed. Furthermore, and probably based on the same difficulties, many studies investigating disability^2^ ^,^ ^3^ ^,^ ^4^, and exercises^5^ ^,^ ^6^ ^,^ ^7^ in HTLV-1 patients with or without HAM/TSP did not use the presence of psychiatric disorders as noninclusion criteria. Based on these facts, the proposed procedure for non-including patients with psychiatric disorders in HTLV-1 studies of pain seems to be unfeasible. Even controlling the results for the presence of these symptoms tends to be difficult, as this analytic approach would fall into low statistical power due to unbalanced groups.
